# Supplementary material for: Empirical methods for controlling false positives and estimating confidence in ChIP-Seq peaks
Source: BMC Bioinformatics. 2008 Dec 5;9:523. doi: 10.1186/1471-2105-9-523 (PMC2628906; doi:10.1186/1471-2105-9-523)
Supplement: Additional File 1 — A variety of html documents from the USeq web site detailing the available applications, their best usage, output file type descriptions, command line menus, etc. [file 1471-2105-9-523-S1.zip › USeqUserGuides/JavaDoc.zip!/JavaDoc/trans/qc/package-summary.html]

trans.qc


|  |  |  |
| --- | --- | --- |
| **Overview** \| **Package** \| Class \| **Use** \| **Tree** \| **Deprecated** \| **Index** \| **Help** | |  |
| **PREV PACKAGE**   **NEXT PACKAGE** | **FRAMES**    **NO FRAMES**     **All Classes** |  |


---

## Package trans.qc

Quality control apps for cel files.

**See:**
  
          **Description**

| **Class Summary** | |
| --- | --- |
| **CelFileQualityControl** | Provides automated qc checking for cel files, flags those of poor quality based on a variety of statistics. |
| **CelFileStats** | Container for cel file and it's associated statistics. |
| **ChartFrame** | Frame for JFree Panels. |
| **CoordinateExtractor1lq** | Extracts control an pm lines from a text 1lq file. |
| **DestypeIntensityCalculator** |  |
| **Parser1lq** | Extracts control an pm lines from a text 1lq file. |
| **StatFlag** | Info related to a particular statistic used to measure a cel file. |

## Package trans.qc Description

Quality control apps for cel files.

---


|  |  |  |
| --- | --- | --- |
| **Overview** \| **Package** \| Class \| **Use** \| **Tree** \| **Deprecated** \| **Index** \| **Help** | |  |
| **PREV PACKAGE**   **NEXT PACKAGE** | **FRAMES**    **NO FRAMES**     **All Classes** |  |


---
